# Two distinct patterns of progression of sporadic cerebral small vessel disease

**DOI:** 10.1177/17474930261459792

**Published:** 2026-06-03

**Authors:** Yutong Chen, Hui Hong, Daniel Tozer, Zihan Sun, Lupei Cai, Hao Li, Anil Man Tuladhar, Frank-Erik De Leeuw, Hugh S Markus

**Affiliations:** 1 Stroke Research Group, Department of Clinical Neurosciences, University of Cambridge, Cambridge, UK; 2 Department of Radiology, The Second Affiliated Hospital of Zhejiang University School of Medicine, Hangzhou, China; 3 Department of Neurology, Research Institute for Medical Research and Innovation, Radboud University Medical Center, Nijmegen, The Netherlands

**Keywords:** Cerebral small vessel disease, trajectory, magnetic resonance imaging, diffusion tensor imaging

## Abstract

**Background:**

Sporadic cerebral small vessel disease (cSVD) is the most common cause of vascular dementia. cSVD features a wide range of neuroimaging markers, including white matter hyperintensity (WMH), lacunes, perivascular spaces, microbleeds, and diffusion magnetic resonance imaging (MRI)-based markers of white matter (WM) injury, and is accompanied by progressive cognitive decline. However, few studies have systematically investigated how and when various imaging and cognitive markers change along the complete cSVD progression trajectory.

**Aims:**

To establish changes in imaging and cognitive markers along the cSVD progression trajectory.

**Methods:**

In this cohort study, we selected 496 participants from the Radboud University Nijmegen Diffusion Tensor and Magnetic Resonance Imaging Cohort (RUNDMC) cohort as the discovery data set. The subtype and stage inference model was used to identify cSVD subtypes and their progression patterns. Five neuroimaging markers (WMH volume, median mean diffusivity (MD) in WM, WM volume, gray matter (GM) volume, and ventricle volume) were used in trajectory construction. The validation cohort was Cambridge cSVD (CamcSVD; n = 330).

**Results:**

We identified two subtypes in RUNDMC and replicated this finding in CamcSVD. One subtype displayed early WMH increase, followed by increased WM MD and tissue volume loss. The other subtype, which we termed “atrophy predominant,” showed early loss of GM and WM and ventricle enlargement, followed by WMH increase and increased WM MD. This subtype displayed an increase in markers of idiopathic normal pressure hydrocephalus (iNPH).

**Conclusion:**

We have delineated two distinct progression patterns in sporadic cSVD. Further studies are needed to investigate the relationship between the atrophy-predominant cSVD subtype and iNPH.

**Data access statement:**

Datasets may be shared upon request to the corresponding author.

## Graphical abstract

**Graphical abstract:**
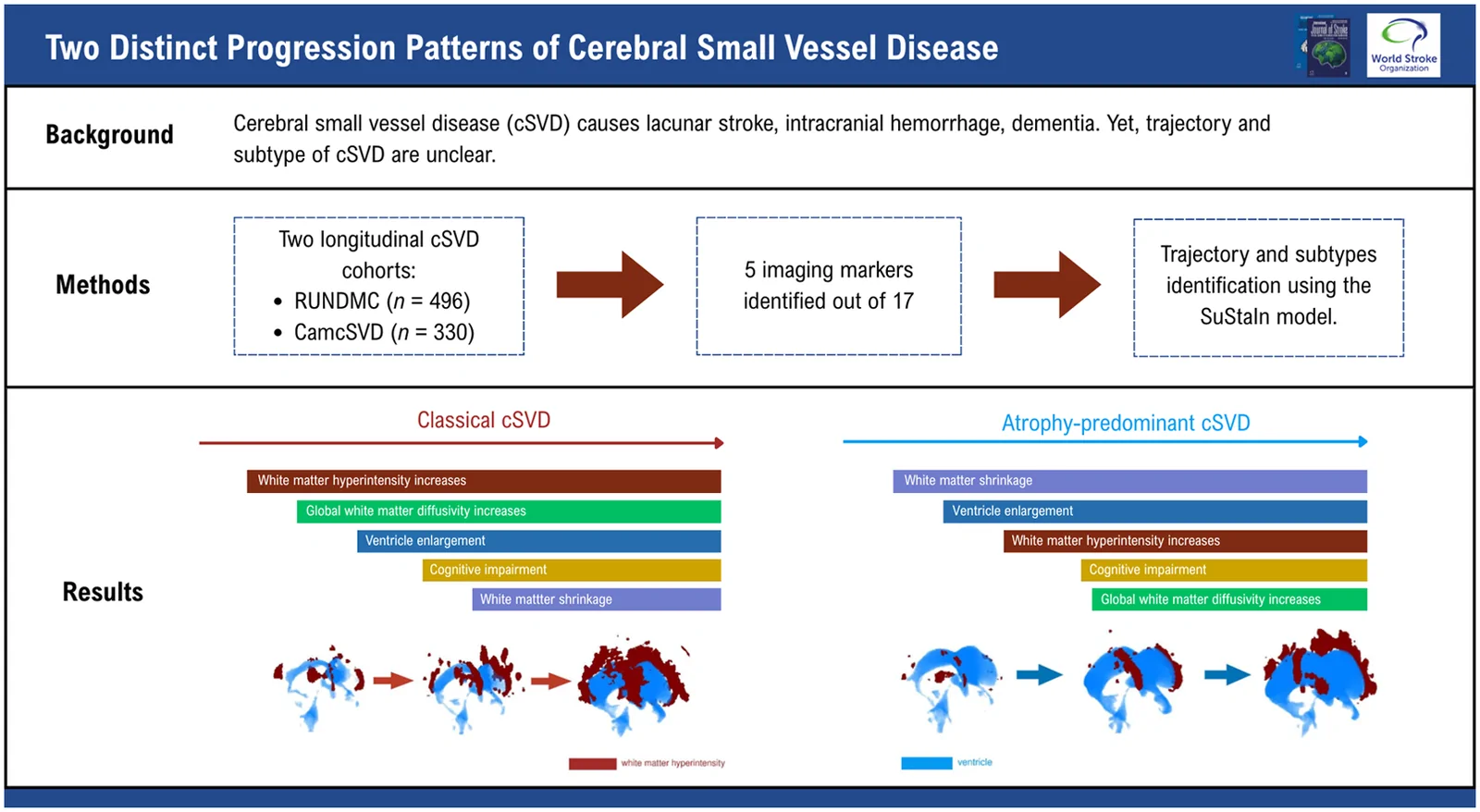

## Introduction

Cerebral small vessel disease (cSVD) causes lacunar stroke and intracerebral hemorrhage and is the most common cause of vascular cognitive impairment and dementia.^1^ Characteristic magnetic resonance imaging (MRI) markers include white matter hyperintensities (WMHs), lacunes, enlarged perivascular space, and microbleeds,^2^ while advanced imaging markers from diffusion MRI (dMRI) show diffuse white matter (WM) ultrastructural damage.^3^ These traditional and advanced imaging markers are increasingly used to quantify disease severity and predict prognosis and to serve as surrogate endpoints for clinical trials of cSVD.^4^ Their usage requires an understanding of the progression patterns of these imaging markers in cSVD.

Traditionally, the trajectory of cSVD is assumed to begin with ultrastructural WM alterations detected by diffusion imaging, followed by structural MRI-based markers such as WMH and brain atrophy, with cognitive impairment representing the final stage.^5,6^ One study focusing on monogenic Cerebral Autosomal Dominant Arteriopathy with Subcortical Infarcts and Leukoencephalopathy (CADASIL) used a knowledge-based approach and reported a similar progression pattern.^7^ However, no study has provided a comprehensive data-driven perspective on the progression pattern of sporadic cSVD. In addition, it is unclear whether there exist cSVD subtypes with different progression trajectories.

Mapping the trajectory of sporadic cSVD progression is challenging, as cSVD-related MRI changes take place over multiple years and even decades. Following up a group of individuals regularly over such a long period with imaging and cognitive testing is difficult owing to financial costs, changes in MRI sequence parameters over time, or participants lost to follow-up. Many studies instead focus only on severe cSVD^8,9^ or mild to moderate cSVD,^10^ or CADASIL patients only.^11^ Lack of a single study that encompasses all stages of cSVD with regular long-term follow-up has limited our ability to construct a timeline of cSVD progression.

One solution is to use a data-driven approach to map participants at different stages of the disease to a common timeline of disease progression.^12^ Participants with milder disease are considered “early” in the timeline, and those with severe disease are considered “late.” It allows for integrating different cohorts of varying cSVD severities without the need for longitudinal follow-up. One common technique to establish this disease timeline is subtype and stage inference (SuStaIn),^13^ a probabilistic model that simultaneously infers the disease subtypes and the trajectory of the subtypes. For each participant, SuStaIn can deduce the most likely disease subtype as well as grade the disease stage for that participant. It has been applied in constructing the trajectories for multiple neurodegenerative diseases.^13,14^

In this cohort study, we used SuStaIn to construct a data-driven trajectory of cSVD progression using five MRI markers in two independent longitudinal cohorts of symptomatic cSVD (Figure 1). Next, we deciphered the temporal sequences of changes in different imaging and cognitive markers. Finally, we investigated whether this approach identified distinct cSVD subtypes with different progression patterns.

**Figure 1.**
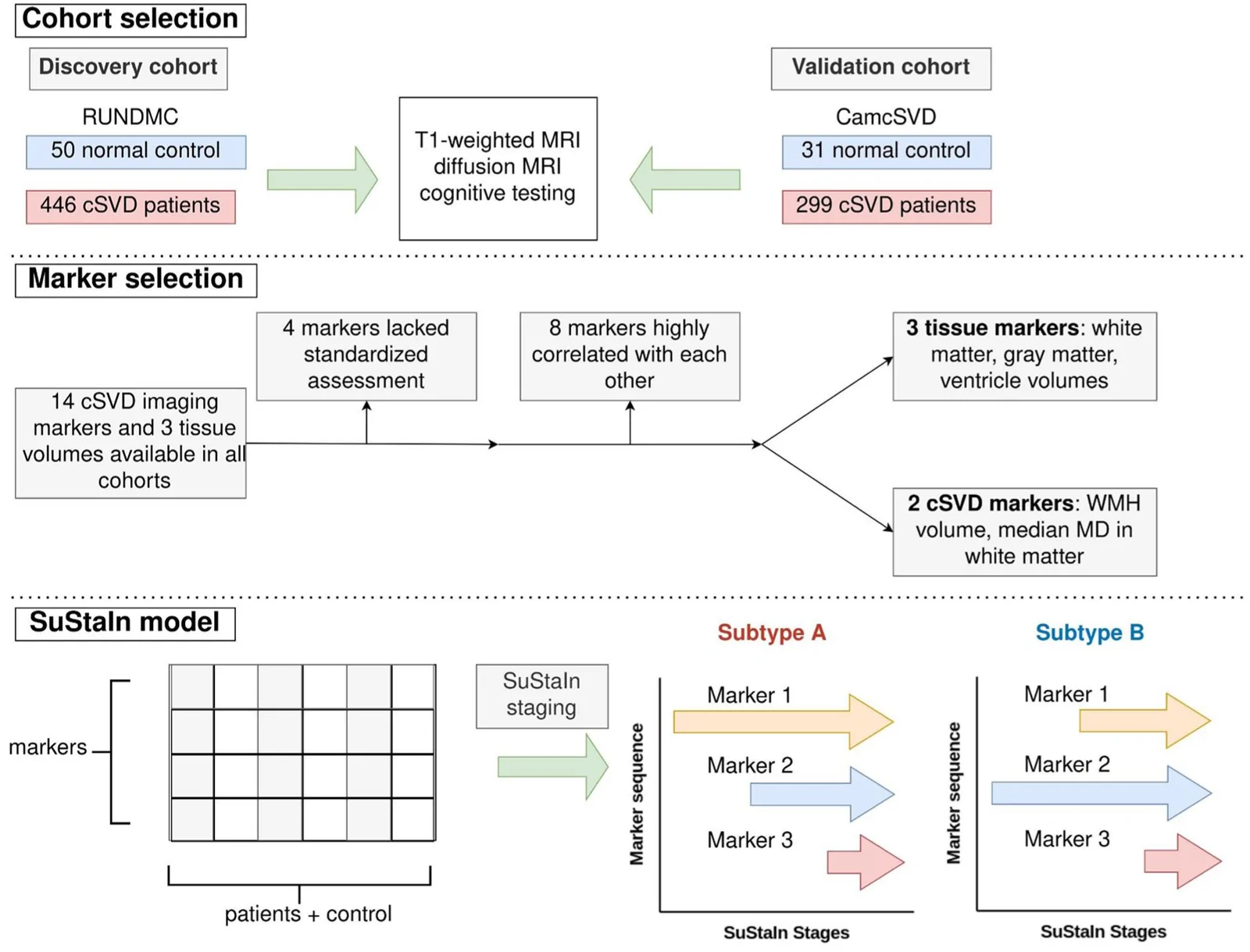
Analysis flowchart. (Cohort selection) Participants underwent T1-weighted MRI, diffusion MRI, and cognitive testing. (Marker selection) Five imaging markers were selected for SuStaIn modeling. (SuStaIn model) SuStaIn simultaneously establishes how markers change in each stage and the subtypes of changes. WMH, white matter hyperintensity; MD, mean diffusivity.

## Materials and methods

### Discovery cohort

The discovery cohort was **R**adboud **U**niversity **N**ijmegen **D**iffusion Tensor and **M**agnetic **R**esonance Imaging **C**ohort (RUNDMC). RUNDMC included participants with lacunes and/or WMH and accompanying stroke, subacute cognitive or motor symptoms.^10^ Among these participants, we selected those with available T1, Fluid-Attenuated Inversion Recovery (FLAIR), and dMRI images.

As RUNDMC lacked a true normal control, we defined a relative normal control group as those with a total cSVD score of zero. This is defined as Fazekas <2, lacune count <1, microbleed count <1, and absence of moderate/severe PVS in the basal ganglia.^15^ As WMH volume >9600 mL has been shown to be equivalent to Fazekas score of ⩾2,^16^ we used WMH <9600 mL to identify those with Fazekas <2. Basal ganglia perivascular space (PVS) count <11 was used as a proxy for lack of moderate/severe PVS.^17^ Participants without microbleeds or lacune count data were excluded.

### Validation cohort

The validation cohort was Cambridge cSVD (CamcSVD), which consists of five symptomatic cSVD cohorts with the same inclusion criteria and similar dMRI sequence parameters (Supplemental Tables S2 and S3). Details of the five individual cohorts making up CamcSVD are given in Supplemental Table S1. The inclusion criteria were as follows: (1) Fazekas grade ⩾2 and (2) clinical lacunar stroke syndrome with an anatomically relevant lacunar infarct either on diffusion weighted imaging for acute infarcts (<3 weeks since onset) or a cavitied lacune on T2 or FLAIR MRI for non-acute infarcts. Exclusion criteria were as follows: any stroke cause other than cSVD (large artery stenosis ⩾50%, cardioembolic source, cortical infarct) and any other cause of WM disease such as demyelination, dementia, and inability to consent. The control group of CamcSVD was derived from the control groups of two of its constituent data sets.^18,19^

In both CamcSVD and RUNDMC, participants were only included if T1, FLAIR, and dMRI scans were available, and cases were excluded if there were corrupted dMRI scans or dMRI motion artifacts.

### Statistics

#### Marker extraction

Imaging parameters for each of the cohorts were detailed in Supplemental Tables S2 and S3. The image preprocessing pipeline for each cohort is detailed in the Supplementary Method section 1.1. Extraction of each imaging and cognitive marker is detailed in the Supplementary Method section 1.2. How each cognitive marker was obtained is detailed in Supplemental Table S4.

#### Cohort characteristics

The Shapiro–Wilk test was employed to evaluate the distribution of continuous variables. Mean and standard deviation are stated for normally distributed variables. Median and interquartile range (IQR) are reported for non-normally distributed variables.

#### Construction of cSVD progression trajectory

Out of 17 cSVD imaging markers, including WMH, PVS, lacunes, and microbleeds, five markers were selected to construct the cSVD trajectory: ventricle volume, WM volume, gray matter (GM) volume, WMH volume, and median mean diffusivity (MD) in WM (Supplementary Results section 2.1 and Supplemental Figure S2 for detailed selection process).

For CamcSVD, dMRI data were harmonized using ComBat^20^ (Supplementary Method section 1.3) with the results in Supplementary Results section 2.2 and Supplemental Figure S3. Preprocessing of each marker is described in Supplementary Method section 1.4.

The SuStaIn model computed the probability of each individual belonging to a particular stage and a particular subtype (Supplementary Method section 1.5). Determination of the optimal number of subtypes is in Supplementary Method section 1.6, Supplementary Results section 2.3, and Supplemental Figure S4. The probability distribution of the participants in each data set was shown in Supplementary Results section 2.4. For each participant, the maximum likelihood SuStaIn stage and the expected stage were calculated. The maximum likelihood stage was the stage where the participant was most likely to be in and was a discrete variable. The expected stage was calculated by the sum of SuStaIn stages weighted by their respective probabilities and was a continuous variable.

#### Validation of cSVD progression trajectory

To validate the cSVD progression trajectory, we compared the optimal number of subtypes and biomarker ordering obtained from the SuStaIn models fitted to each data set. Biomarker ordering was visualized using positional variation diagrams, which displayed the most likely stages where biomarkers reach particular z-score thresholds. We calculated the expected value of the stage when a biomarker reached a particular z-score and compared this result across the three data sets using Pearson correlation.

Next, the staging and subtype consistency of the same participants were studied using the longitudinal follow-up visits in the CamcSVD (1, 2, and 3 years after baseline visit) and RUNDMC data sets (5 and 9 years after baseline visit). For staging consistency, a linear regression model was fitted between the time of visit and the expected stages of each visit for every participant. Due to the progressive nature of cSVD, a high staging consistency means that the expected stages remain stable or increase over time, that is, the slope of linear regression would not be significantly different from 0 or would be positive. For subtype consistency, the proportion of patients who had stable subtype assignment was calculated. This was defined as those whose subtype assignment did not change across the follow-up visits, or those whose maximum likelihood stage at baseline is 0, which corresponds to “normal” subjects in a SuStaIn model.^14^ Subtype assignment probabilities were compared between the participants with stable subtypes and those without, using the Wilcoxon rank sum test.

#### Sequence of imaging and cognitive markers along cSVD trajectories

The SuStaIn stages with fewer than 10 participants were excluded from the analysis. To identify which marker exhibits early or late changes, the mean z-score was calculated for all individuals assigned to the maximum likelihood SuStaIn stage. A marker was considered to exhibit “earlier” change than another marker if it took fewer SuStaIn stages to achieve a mean z-score of 1. If two markers achieved mean z-scores of 1 at the same SuStaIn stage, then we compared the stages in which mean z-scores of 2 were reached.

#### Comparison of subtypes of cSVD progressions

Participants with subtype assignment probability ⩽0.8 were excluded. Participants with missing values for a particular marker were excluded from the analysis pertaining to that marker, with the degree of missingness indicated in Supplemental Table S5. A linear regression model was constructed with the marker being the outcome and the subtype being the independent variable. For binary variables (e.g. diabetes or hypertension status), logistic regression was used. The model was corrected for age and sex. For cognitive outcomes, the model was corrected for age, sex, and years of education. To assess differences in cerebrospinal fluid (CSF) dynamics between the two subtypes, we obtained diffusion tensor imaging along perivascular space (DTI-ALPS),^21^ and the model was corrected for age, sex, and median MD in WM. To compare PVS volumes in the CamcSVD data set, given the differing MRI sequence parameters (Supplemental Table S2), we only tested the comparison within one of the cohorts, the St George’s Cognition and Neuroimaging in Stroke (SCANS) cohort. The standardized coefficient and *p* value associated with subtype B were reported. *p* Values were adjusted by the Benjamini–Hochberg method.

Given the overlap of the clinical and radiological manifestations between cSVD and iNPH,^22^ we also investigated whether cSVD subtypes exhibited markers of idiopathic normal pressure hydrocephalus (iNPH). We derived T1-weighted MRI-based markers for iNPH using methods modified from a previous article (Supplemental Figure S1).^23^

#### Sensitivity analysis

To investigate whether the SuStaIn model was influenced by aging processes that were not specific to cSVD, we performed a sensitivity analysis, excluding participants demonstrating significant cortical atrophy or ventriculomegaly. This was defined as participants in the bottom 10% of normalized GM volume or the top 10% of normalized ventricle volume. SuStaIn modeling was repeated on the remaining subset. The correlation between the original staging and the staging during sensitivity analysis was calculated using Spearman correlation. Similarly, we evaluated the correlation of biomarker ordering. To quantify the concordance of subtype assignment between the original and sensitivity analyses, we performed McNemar’s test.

To investigate the specificity of cSVD for participants in each SuStaIn stage, we calculated the total cSVD score for each participant.^15^ As WMH volume >9600 mL has been shown to be equivalent to Fazekas score of ⩾2,^16^ we used WMH >9600 mL to identify those with Fazekas ⩾2.

#### Software

Image preprocessing was implemented in Python 3.9. SuStaIn modeling utilizes the *pySusStaIn* package. Statistical analyses were performed in R 4.5.0. The source code will be available (https://github.com/Yutong441/DTItime).

## Results

### Cohort characteristics

In the RUNDMC validation cohort, we studied 496 participants with 1131 scans. The CamcSVD validation cohort contained 330 participants with a total of 643 scans. The demographics, imaging, and cognitive features of RUNDMC and CamcSVD were shown in Supplemental Table S6.

### Identification of cSVD progression trajectories

We identified two distinct subtypes of cSVD progression in RUNDMC (Supplemental Figure S4, Supplementary Results section 2.2). Subtype A accounted for 50% of the participants, and subtype B 50%. For each subtype, we delineated 15 stages of cSVD progression.

In subtype A, WMH volume and cognitive markers displayed the earliest changes (Figure 2), followed by increases in median MD. Late markers were tissue volumes (decreased GM, decreased WM, and increased ventricle volume) and cognition. In subtype B, cognitive markers and GM and ventricle volumes displayed the earliest changes (Figure 2), followed by WMH volume increase. Diffusion and cognitive markers displayed later changes. Although GM volume decreased at stage 1, it plateaued until stage 11 before further decline. Examples of FLAIR images in each stage of the trajectory are in Figure 3, and we summarized the main events in each subtype of cSVD progression in Figure 4.

**Figure 2.**
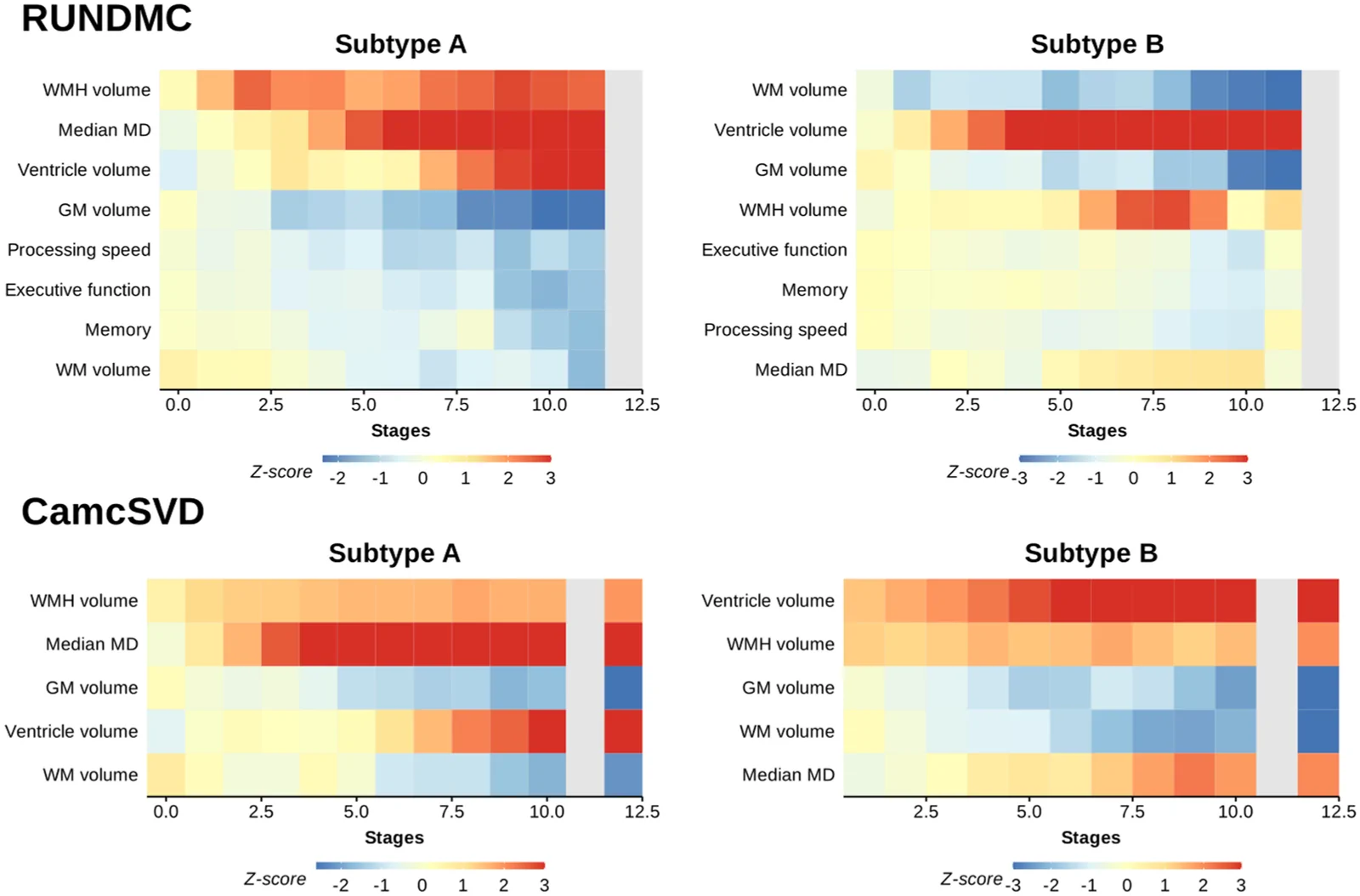
Progression pattern for each marker for subtypes A and B in each data set. Gray colors are used in the stages with fewer than 10 participants. Each row shows the mean z-scores of a marker in each SuStaIn stage. Z-scores have been capped between −3 and 3. For cognitive markers, lower values mean worse cognition. Cognitive changes in the CamcSVD data set could not be assessed, as different cohorts within the CamcSVD data set had different cognitive tests. GM, gray matter; WM, white matter; WMH, white matter hyperintensity.

**Figure 3.**
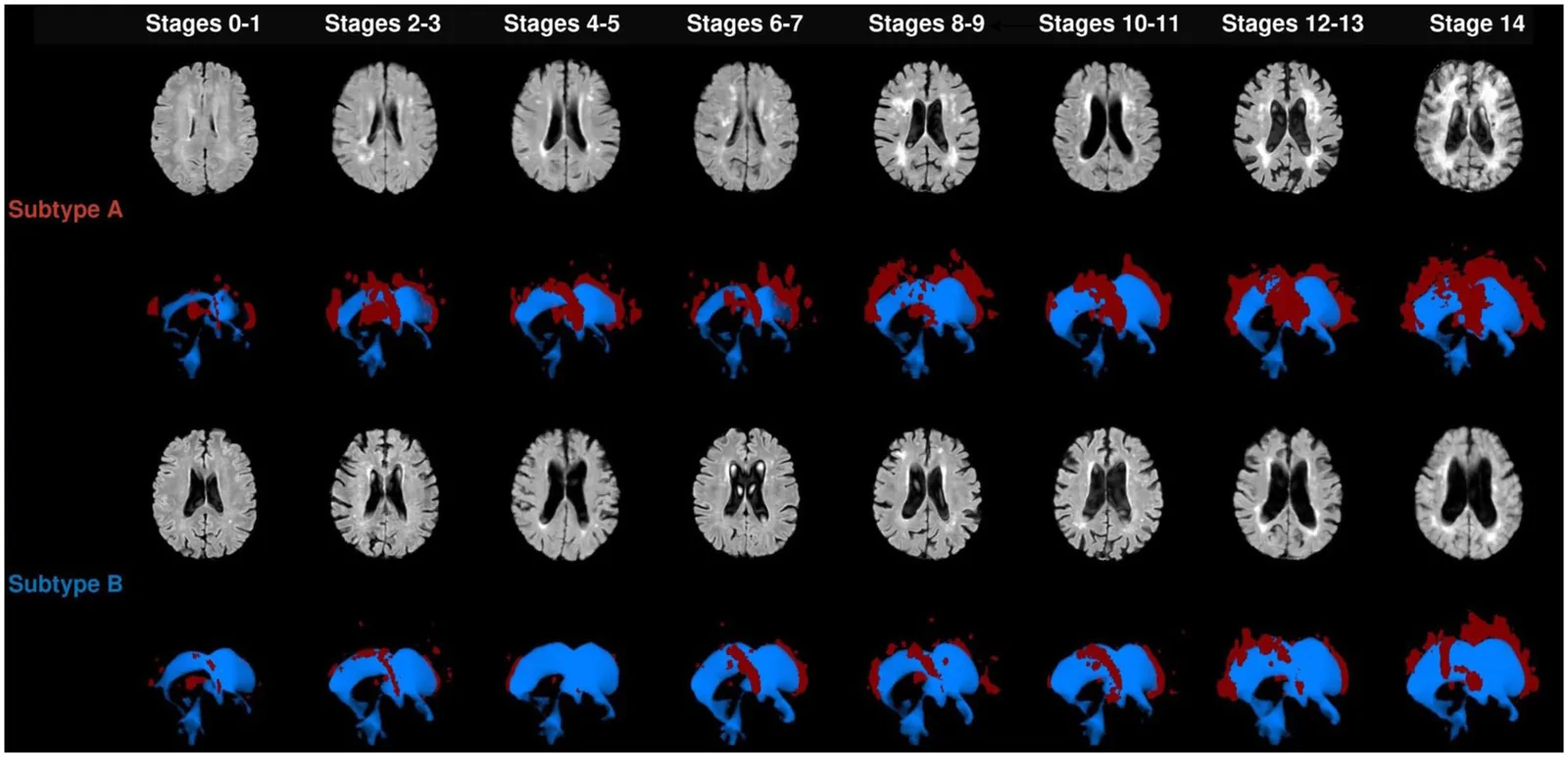
Examples of FLAIR images and three-dimensional (3D) reconstruction of the ventricles and WMH of the participants in the RUNDMC cohort along different stages of cSVD. Color coding for 3D reconstruction: red: WMH and blue: ventricle. WMH, white matter hyperintensity.

**Figure 4.**
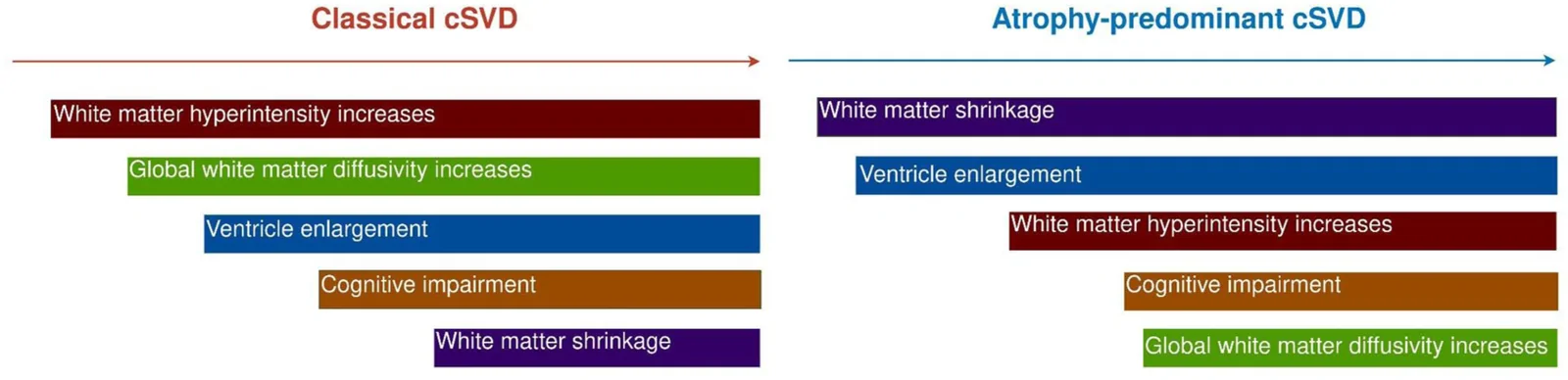
Summary of progressions of two cSVD subtypes.

### Validation of cSVD progression trajectories

In the validation cohorts, we found the same two distinct cSVD subtypes in the CamcSVD data set, with subtype A accounting for 73% of the participants. The same sequence of imaging and cognitive changes (Figure 2 and Supplemental Figure S5) was observed in both subtypes as in RUNDMC, and the Pearson correlation of the stages of imaging marker changes between RUNDMC and CamcSVD was 0.9 for subtype A and 0.79 for subtype B. (Supplemental Figure S6).

We next investigated the consistency of staging using the longitudinal follow-up data in the CamcSVD and RUNDMC cohorts. In RUNDMC and CamcSVD, in 70.9% and 96.1% of the participants, respectively, the assigned stage either increased or remained constant (Supplemental Figure S7). In terms of subtype consistency, the proportion of participants with stable subtype assignment across different visits was above 94% in both CamcSVD and RUNDMC cohorts (Supplemental Figure S8).

### Comparison of subtypes of cSVD progression

In linear regression analysis, compared with subtype A, no difference in age (β = 0.10 [95% CI, −0.10 to 0.30], *p* = 0.173) or the level of education was noted (β = 0.10 [95% CI, −0.00 to 0.30], *p* = 0.078). Subtype B tends to have fewer cases of hypertension (β = −0.60 [95% CI, −0.90 to 0.20], *p* = 0.005) and active smoking (β = −0.30 [95% CI, −0.40 to 0.10], *p* = 0.016; Figure 5).

**Figure 5.**
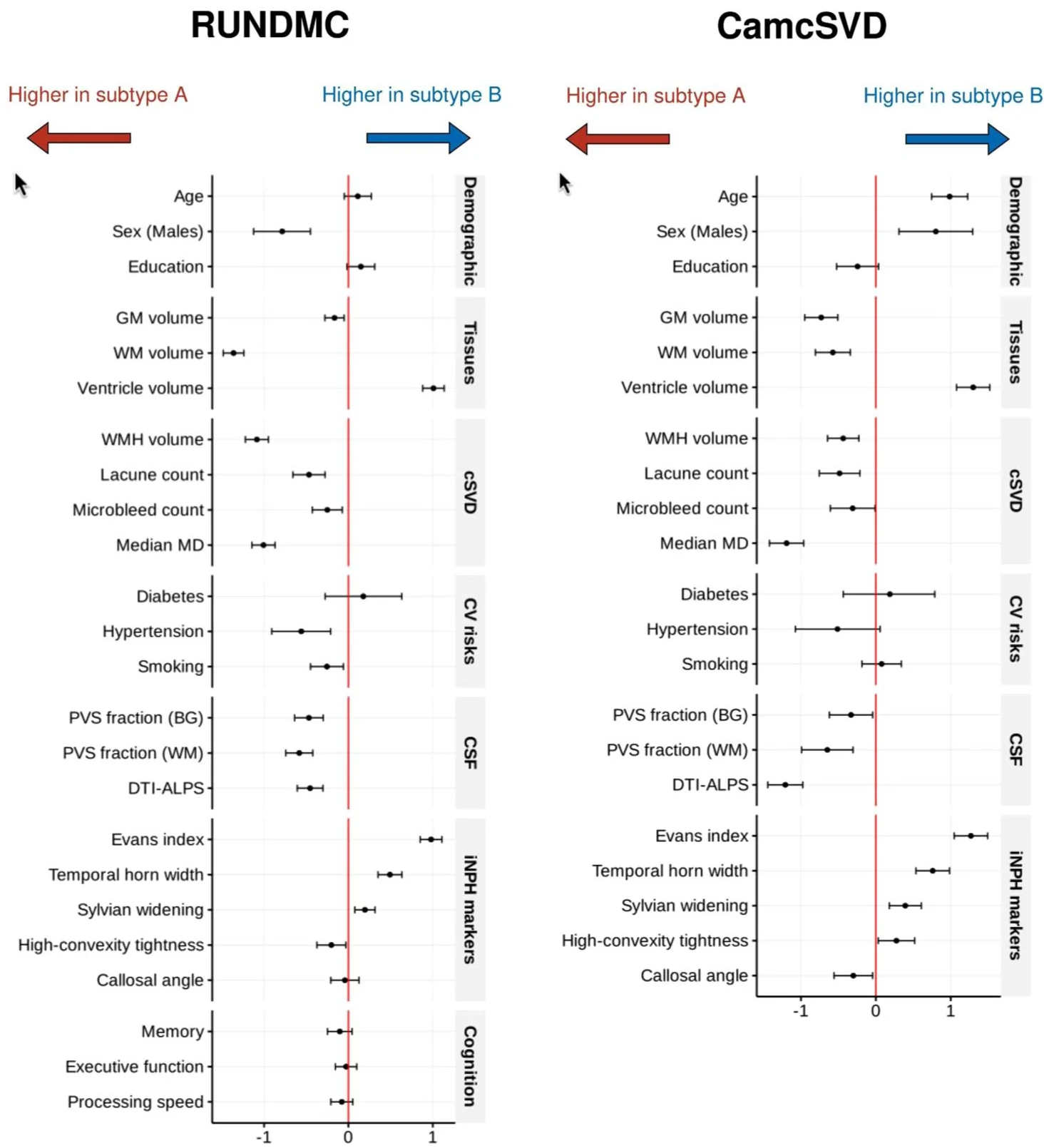
Regression coefficients (*x*-axis) between SuStaIn subtype and each demographic, imaging, and clinical markers. For a marker, a positive coefficient indicates a higher value of this marker in subtype B than in subtype A. Error bars represent 95% confidence intervals. For cognitive markers, lower values mean worse cognition. GM, gray matter; WM, white matter; WMH, white matter hyperintensity; MD, mean diffusivity; CV, cardiovascular; iNPH, idiopathic normal pressure hydrocephalus.

Subtype B showed significantly reduced GM volume (β = −0.20 [95% CI, −0.30 to −0.10], *p* = 0.005), WM volume (β = −1.40 [95% CI, −1.50 to 1.20], *p* < 0.001), and higher ventricular volume (β = 1.00 [95% CI, 0.90–1.10], *p* < 0.001; Figure 5). Subtype B had lower levels of cSVD markers including WMH volume (β = −1.10 [95% CI, −1.20 to 0.90], *p* < 0.001), lacune count (β = −0.50 [95% CI, −0.70 to 0.30], *p* < 0.001), microbleed count (β = −0.30 [95% CI, −0.40 to 0.10], *p* = 0.006), and median MD values (β = −1.00 [95% CI, −1.10 to 0.90], *p* < 0.001).

We further examined whether markers of CSF dynamics differed between the two groups. Subtype B had lower DTI-ALPS (β = −0.50 [95% CI, −0.60 to 0.30], *p* < 0.001). In contrast, subtype A had more enlarged PVS in both basal ganglia (β = −0.50 [95% CI, −0.60 to 0.30], *p* < 0.001) and WM regions (β = −0.60 [95% CI, −0.70 to 0.40], *p* < 0.001).

Given the enlarged ventricles in subtype B compared with subtype A, we compared the iNPH markers between the two subtypes. Subtype B showed significantly higher Evans index (β = 1.00 [95% CI, 0.90–1.10], *p* < 0.001), larger width of the temporal horn of the lateral ventricles (β = 0.50 [95% CI, 0.40–0.60], *p* < 0.001), wider Sylvian fissure (β = 0.20 [95% CI, 0.10 to 0.30], *p* = 0.002), and lower high-convexity tightness (β = −0.20 [95% CI, −0.40 to 0.00], *p* = 0.027). No differences in callosal angles (β = −0.00 [95% CI, −0.20 to 0.10], *p* = 0.625) were found. Subtype B contained three times more participants with radiologically probable iNPH than subtype A (RR = 3.18 [95% CI, 2.25–4.49], *p* < 0.001, Supplemental Table S7). In Figure 5, similar differences were observed in CamcSVD regarding the pattern of cSVD imaging markers and the iNPH imaging markers.

### Sensitivity analysis

After excluding participants with significant GM atrophy and/or ventriculomegaly, 384 out of 496 participants from RUNDMC and 273 out of 330 participants from CamcSVD remained. Two subtypes of cSVD were identified from both cohorts in the sensitivity analysis (Supplemental Figure S9, Supplementary Results Section 2.3). The correlation between the original SuStaIn stages and the stages in sensitivity analysis was 0.96 in RUNDMC and 0.99 in CamcSVD (Supplemental Figure S10). As for the staging of the imaging biomarkers, the correlation was 0.96 and 0.78 for subtypes A and B in RUNDMC, and 0.98 and 0.92 for subtypes A and B in CamcSVD (Supplemental Figure S11). Regarding the stability of subtype assignment, the accuracy was 0.92 (*p* < 0.001) for RUNDMC and 0.98 (*p* = 0.003) for CamcSVD (Supplemental Table S8).

To investigate the specificity of cSVD for participants in each SuStaIn stage, we plotted the total cSVD scores along the SuStaIn stages. We found that in both RUNDMC and CamcSVD, participants in the early stages exhibited total cSVD scores with a median between 1 and 2, which rose to a median between 3 and 4 after stage 11 (Supplemental Figure S12).

## Discussion

Using a data-driven approach, we have described a timeline of sporadic cSVD progression and identified two distinct patterns of disease progression.

In subtype A, WMH volume showed the earliest increase, followed by diffusion MRI markers, while the rate of increase in diffusion markers outstripped that of WMH between SuStaIn stages 2–5. The initial lag of global diffusion metrics may be because, in these markers, early small regional WM changes are masked by the remaining healthy WM tissues. Compared with cSVD imaging markers, GM and WM losses occurred at a later stage, which could result from retrograde degeneration secondary to WM damage and tract disruptions.^24^

In subtype B, early WM loss and ventricle enlargement were followed by WMH and median MD increase. The WMH volumes and median MD in subtype B were smaller than those in subtype A, suggesting subtype B is an atrophy-predominant subtype. Although ventricular enlargement could be driven by tissue loss, subtype B has a higher-convexity tightness than subtype A, a marker of iNPH that cannot be attributed to global GM atrophy. Furthermore, subtype B has a higher prevalence of radiologically probable iNPH. The overlap of cSVD and iNPH is plausible given that both conditions share common risk factors, such as aging and vascular comorbidities,^25^ as well as similar clinical features, including cognitive impairment and gait disturbance.^26^ Furthermore, cSVD imaging markers are frequently observed in iNPH; a recent study reported that 78% of iNPH patients exhibited at least one cSVD imaging feature.^22^ However, given the lack of iNPH confirmatory tests, such as CSF shunt response in our study cohorts, this study could not definitely conclude whether this atrophy-predominant subtype of cSVD is underlined by iNPH. Future studies could investigate whether cSVD patients of this subtype respond to CSF shunting.

In both subtypes, we found that cognitive declines were preceded by dMRI markers changes, consistent with previous studies that showed dMRI markers can predict cognitive decline and/or development of dementia.^27,28^

One emerging mechanism in cSVD is dysfunction of CSF dynamics and transport.^29^ The recent development of DTI-ALPS^21^ enables quantification of CSF dynamics by utilizing diffusion MRI to assess the diffusion of water molecules along the perivascular space of the deep medullary veins. Our results showed that cSVD subtype B had lower DTI-ALPS and lower PVS volumes compared with subtype A. As DTI-ALPS was more strongly correlated with venous disruption than PVS volume^30^ and venous insufficiency is known to be involved in cSVD pathogenesis,^31^ it was possible that venous insufficiency plays a greater role in CSF dynamics disruption in subtype B than subtype A. In contrast, subtype A was characterized by higher PVS volume in both basal ganglia and WM regions. As PVS enlargement reflects arterial dysfunction and CSF stagnation,^32^ this suggests arterial damage may be a more important cause of CSF dynamics dysfunction in cSVD subtype A. Our results highlight the heterogeneity of CSF dynamics dysfunctions in cSVD pathogenesis, suggesting the two cSVD subtypes may require different treatment approaches.

The strength of the study is that we have applied an unbiased data-driven methodology to infer the trajectory of cSVD progression. We have systematically assessed structural and diffusion MRI markers, cardiovascular risk factors, and cognitive markers in CamcSVD and RUNDMC with a total of 826 participants.

One limitation is that we were unable to include certain cSVD imaging markers, including PVS volume, microbleed, and lacune counts in trajectory construction. Inability to include PVS volume was due to a discrepancy in MRI field strengths among the CamcSVD cohorts, leading to inconsistencies in estimating PVS volume. Inability to include microbleed and lacune counts was because of the inability to perform a z-transform for these two ordinal variables, which was a requirement to perform SuStaIn modeling. Future research should focus on developing a trajectory inference tool independent of z-scoring. The second limitation is that although we found harmonization of diffusion metrics within the CamcSVD data set reduced cohort effects, the harmonization process may be incomplete. This could potentially influence event ordering in the CamcSVD data set. The third limitation is the discrepancy in demographics between the discovery (RUNDMC) and validation (CamcSVD) cohorts. The CamcSVD cohort featured more severe cSVD markers and more vascular risk factors. This discrepancy limited the assessment of the generalizability of the trajectories constructed by SuStaIn models and could explain why the biomarker ordering was highly similar but not identical between the two data sets.

Furthermore, as the prevalence of non-vascular pathology in cSVD patients can be up to 70%,^33^ disentangling vascular from non-vascular pathology is challenging. Therefore, we have applied a stringent selection process in our cohorts, and our sensitivity analysis has shown that removing participants with atrophy and ventriculomegaly did not influence the results significantly. However, we cannot fully eliminate non-vascular processes, and the presence of a subtype containing iNPH-like imaging features may indicate it is potentially influenced by non-vascular pathology.

Finally, our study has been limited by the selection of early-stage cSVD patients. This is partly because, to ensure the specificity of our cohorts, we have used a stringent definition of including patients with lacunar infarcts and/or confluent WMH with Fazekas⩾2. Another reason is the difficulty of verifying that a low burden of WMH is of vascular origin.^34^ Therefore, instead, we have incorporated normal control to approximate the early end of the disease spectrum. Future studies could consider applying a combination of advanced imaging techniques, including cerebral vascular reactivity and dynamic contrast-enhanced MRI, to assess blood–brain barrier leakage associated with endothelial damage, to evaluate the brain small vessel injury directly, and to include more participants of early-stage cSVD.

In conclusion, we have provided a timeline of changes in cSVD from early to late stages. We have delineated two distinct progression patterns in sporadic cSVD and identified the sequential pattern of progression of imaging changes in each. The second subtype shares imaging markers with iNPH, requiring further investigations of its pathogenesis.

## Supplemental Material

sj-docx-1-wso-10.1177_17474930261459792 – Supplemental material for Two distinct patterns of progression of sporadic cerebral small vessel disease Supplemental material, sj-docx-1-wso-10.1177_17474930261459792 for Two distinct patterns of progression of sporadic cerebral small vessel disease by Yutong Chen, Hui Hong, Daniel Tozer, Zihan Sun, Lupei Cai, Hao Li, Anil Man Tuladhar, Frank-Erik De Leeuw and Hugh S Markus in International Journal of Stroke

## Data Availability

Data sets may be shared upon request to the corresponding author.
