# Supplementary material for: Two distinct patterns of progression of sporadic cerebral small vessel disease
Source: Int J Stroke. 2026 Jun 3;21(9):1348–57. doi: 10.1177/17474930261459792 (PMC13672929; doi:10.1177/17474930261459792)
Supplement: Supplementary file 1 [file sj-docx-1-wso-10.1177_17474930261459792.docx]

# Two distinct patterns of progression of sporadic cerebral small vessel disease

## 1. Supplementary methods

### 1.1 Image preprocessing

In the CamcSVD and RUNDMC datasets, for T1 and FLAIR images, the mrtrix package was used to remove Gibbs artifacts and the ANTs package was used to correct magnetic field non-homogeneity.[^1^](https://www.zotero.org/google-docs/?R09Hgo) T1 images were skullstripped using the HD-BET package.[^2^](https://www.zotero.org/google-docs/?wjTOl8) FLAIR images were registered to T1 images via rigid registration. The brain masks from T1 images were applied to remove the skulls from FLAIR images.

Diffusion MRI (dMRI) data was corrected for eddy current using either eddy_correct function from FSL (MINERVA, NETWORKS, and SCANS cohorts) or the eddy function (PRESERVE and RUNDMC cohorts). For the datasets that were processed by the eddy command, DTI images were first denoised using the dwidenoise function from the mrtrix package, and Gibbs artifacts were removed. Motion correction was carried out on the DWI images using the mcflirt function from FSL. The b0 volumes were averaged. The mean b0 volume and the corresponding skullstripped T1 image were used to synthesize the reverse phase encoding DTI image.[^3^](https://www.zotero.org/google-docs/?CRI65k) The reversed encoding and original DTI can be used in the eddy command.

### 1.2 Marker extractions

#### **1.2.1 Tissue volumes**

FreeSurfer was used to parcellate the T1 images.[^4^](https://www.zotero.org/google-docs/?5HFVbn) Ventricle volume was the sum of the volumes of left lateral, right lateral, third and fourth ventricles (FreeSurfer labels 4, 5, 43 and 44 respectively). Gray matter volume was the sum of cortical and subcortical gray matter volumes (FreeSurfer labels 8, 10, 11, 12, 13, 17, 18, 26, 28, 47 49, 50, 51, 52, 53, 54, 58, and 60). White matter volume was the sum of cerebral, cerebellar and brainstem white matter volumes (FreeSurfer labels 2, 7, 16, 41 and 46). Intracranial volume (ICV) was the sum of gray matter, white matter, ventricle and cerebrospinal fluid (CSF) volume (FreeSurfer label 24). All volume measures were normalized by dividing by ICV.

#### **1.2.2 cSVD markers**

White matter hyperintensity (WMH) was segmented by by Hypermapper in CamCSVD and RUNDMC.[^5^](https://www.zotero.org/google-docs/?2KaSMl) WMH volume was divided by ICV and log-transformed. Mean diffusivity (MD) and fractional anisotropy (FA) maps were obtained using FSL *dtifit* command,[^6^](https://www.zotero.org/google-docs/?cGmIKU) using the 1000 mm^2^/s shell. To calculate median MD and FA in white matter region, pseudo-T2 images were created from dMRI[^7^](https://www.zotero.org/google-docs/?EI6A08) and parcellated by FreeSurfer into gray matter, white matter and CSF. Median values for each of the dMRI maps were calculated in the white matter regions. Peak width of skeletonized mean diffusivity (PSMD) was calculated from the MD map.[^8^](https://www.zotero.org/google-docs/?I83XN9) Lacunes and microbleeds were manually counted.

#### **1.2.3 Cardiovascular risk factors**

Cardiovascular risk factors in the RUNDMC and CamcSVD datasets were obtained from self-report questionnaires, including hypertension, diabetes and status of smoking. Smoking status was encoded as “0”: never-smoker, “1”: ex-smoker, “2”: current smoker.

#### **1.2.4 CSF dynamic markers**

Given the importance of CSF dynamic dysfunction in the pathogenesis of cSVD,[^9,10^](https://www.zotero.org/google-docs/?0kb2X5) we evaluated two CSF dynamics markers: perivascular space (PVS) volume and diffusion tensor imaging along perivascular space (DTI-ALPS). PVS was automatically segmented using a deep learning model.[^11^](https://www.zotero.org/google-docs/?rsHo9u) In the CamcSVD cohort, given the different magnetic field strengths of the MRI scanners (**Table S2**), PVS segmentation was only performed in the SCANS cohort. PVS volume was quantified in the basal ganglia and white matter regions. Basal ganglia and white matter segmentations were derived from FreeSurfer.[^4^](https://www.zotero.org/google-docs/?niHuu6) DTI-ALPS was calculated as described previously.[^12^](https://www.zotero.org/google-docs/?FlmGZo)

#### **1.2.5 Idiopathic normal pressure hydrocephalus (iNPH) markers**

Given the overlap of the clinical and radiological manifestations between cSVD and iNPH,[^13^](https://www.zotero.org/google-docs/?WxZsZB) we derived T1-weighted MRI-based markers for iNPH using methods modified from a previous paper.[^14^](https://www.zotero.org/google-docs/?ktvD8O) These markers were Evans index, temporal horn width, Sylvian widening, high convexity tightness and callosal angle (**Figure S1**).

Preprocessed T1-weighted MRI images were parcellated using FreeSurfer.[^4^](https://www.zotero.org/google-docs/?9cXUk1) The T1 images were registered to the MNI space using rigid registration. Next, anterior commissure and posterior commissure were automatically detected.[^15^](https://www.zotero.org/google-docs/?rMB7ZZ) The T1 images were rotated such that the horizontal plane passes through both the anterior and posterior commissures. The parcellation masks were warped to the same space as the T1 images. All subsequent calculations were performed in this space.

The Evans index was defined as the ratio between the maximum distance between the frontal horns of the lateral ventricles and the maximum distance of the intracranial volume on the same axial plane. To calculate the Evans index, first, corpus callosum was automatically detected and parcellated using the Witelson method.[^15^](https://www.zotero.org/google-docs/?WO8Ywm) Frontal horn was defined as the voxels in the lateral ventricle (FreeSurfer labels 4 and 43) anterior to the most posterior voxel of the rostrum of the corpus callosum. Maximum horizontal distance in this frontal horn mask was divided by the maximum horizontal distance in the intracranial mask generated by FreeSurfer to obtain the Evans index (**Figure S1A**).

The width of the temporal horn was calculated based on the temporal horn segmentation from FreeSurfer labels 5 and 44 of the parcellation mask. Each temporal horn was divided into a set of connected segments. Connected segments with fewer than 10 voxels were discarded. Among the remaining segments, the most anterior segment was chosen (**Figure S1B**). The volume (V) and the length (l) of the long-axis of the segment was calculated using scikit-image package.[^16^](https://www.zotero.org/google-docs/?5zQjev) Assuming that temporal horn has a cylindrical shape, the mean width of the temporal horn (d) was calculated via the following equation:

$d=2\sqrt{}$ .

The degree of Sylvian widening was measured by selecting the cortical regions adjacent to the Sylvian fissures: FreeSurfer labels 1030, 1031, 1034, 1035, 2030, 2031, 2034, and 2035. For each Sylvian fissure, the largest connected component was extracted and a 3D convex hull was fitted to this component. For the intracranial tissues inside this convex hull, the percentage of voxels labelled as CSF (FreeSurfer label 24) was used to indicate Sylvian widening (**Figure S1C**).

The degree of high-convexity tightness was measured by selecting the upper three-quarters of the intracranial region between 2 peaks of the lateral ventricle. For the intracranial tissues inside this region, the percentage of voxels not labelled as CSF (FreeSurfer label 24) was used to indicate high-convexity tightness (**Figure S1D**).

The callosal angle was defined as the angle of the corpus callosum in the coronal image perpendicular to the anterior/posterior commissure plane at the posterior commissure. To calculate the callosal angle, the coronal plane at the posterior commissure was selected. For each lateral ventricle in this plane, the most medial point was selected. Voxels below this medial point were set to 0. The highest voxel was identified and the voxels lateral to the highest voxel were set to 0. Among the remaining voxels, a minimum enclosing triangle was calculated. The callosal angle was calculated as the angle between the 2 triangles (**Figure S1E**).

Using these markers, we identified participants with radiologically probable iNPH according to modified International Guidelines for clinical diagnosis of iNPH:[^17^](https://www.zotero.org/google-docs/?fVoKXn) 1) Evans index > 0.3, and 2) no discernible CSF flow obstruction and 3) enlargement of temporal horn (width ≥6mm) or periventricular signal changes not attributable to microvascular ischemic change or demyelination or callosal angle < 90^0^.

#### **1.2.6 Cognitive markers**

In RUNDMC, participants underwent cognitive assessments to evaluate their memory, executive function, and processing speed (**Table S4**). In CamcSVD, cognitive performance was assessed using different cognitive tests.[^18^](https://www.zotero.org/google-docs/?k1kj3C) Due to the difficulty of standardizing cognitive measures across the 5 constituent datasets, the cognitive features were not analyzed in the CamcSVD dataset.

### 1.3 Data harmonization

To reduce the cohort effects that arise from the differences in scanning sequence and/or processing techniques, ComBat harmonization was applied on the imaging data in the discovery and replication cohorts. Among the 6 features used in the SuStaIn model, 4 were derived from structural MRI and 2 from diffusion MRI (dMRI). The 4 structural metrics (white matter hyperintensity [WMH] volume, white matter volume, gray matter volume and ventricle volume) were derived from a similar set of analysis pipelines (see main methods section) and harmonization was not necessary. ComBat harmonization[^19^](https://www.zotero.org/google-docs/?0Acbws) was applied on the two dMRI metrics–peak width of skeletonized mean diffusivity (PSMD) and median mean diffusivity (MD) in white matter. Harmonization was performed across all the cohorts in the CamcSVD dataset (SCANS, PRESERVE, NETWORKS and MINERVA), as they share similar sequence acquisition parameters (**Table S2-3**) and inclusion criteria.

We followed Chen *et al.* (2023)[^20^](https://www.zotero.org/google-docs/?uXWdvT) in assessing whether ComBat harmonization preserved biological variability while reducing cohort effects. Biological variability is defined as the proportion of variance explained by age, sex, and WMH volume. This was calculated from the R^2^ of the linear regression between each dMRI metric and age, sex, and WMH volume. To assess the impact of ComBat on cohort effects, homogeneity of variance was computed between the variance of each dMRI metric before and after harmonization using Bartlett’s test. Statistical significance of Bartlett’s test indicates that for a particular metric, different datasets exhibit different variances, i.e., existence of cohort effects. To evaluate cohort effects along the principal components, principal component analysis (PCA) was computed using the 2 dMRI markers and the 4 structural markers. As an additional method of assessing cohort effects, the cohort prediction test was performed. This involved constructing a support vector machine of a radial kernel to predict which cohort each subject belongs to, using the 6 MRI markers. High prediction accuracy from 10-fold cross validation indicates the existence of cohort effects.

### 1.4 Preprocessing of each marker

Each marker is linearly adjusted for age and sex, using the slopes and intercepts obtained by fitting a linear regression model in the normal control population of each dataset. Each marker is z-transformed, using the mean and standard deviation derived from the normal control. Age and sex adjustment and z-transform were carried out independently in each of the 3 datasets. If the value of a marker is lower in cSVD patients than normal control, the sign of the marker was flipped, so that for all markers, higher values were found in cSVD. The z-scores of each imaging marker were capped at 10.

### 1.5 SuStaIn setting

Subtype and stage inference was performed by SuStaIn, which combined disease clustering with disease progression modeling. We applied a linear z-score SuStaIn model, where each stage is characterized by a particular imaging marker reaching a particular threshold of z-score. Three thresholds of z-scores were used: 1, 2 and 3. With 6 imaging markers, there were 6 x 3 = 18 stages in total. Model uncertainty was estimated by performing 100,000 Markov chain Monte Carlo iterations.

### 1.6 Selection of the optimal number of subtypes

The optimal number of subtypes in each dataset was determined by 10-fold cross-validation. SuStaIn models were fitted for 1, 2, 3, 4 or 5 subtypes. Each SuStaIn model was fitted to 90% of the data and log likelihood of fitting was calculated on the remaining 10% data that was not used during training. Comparison of log likelihood was made using the Wilcoxon rank sum test. For each model, cross information validation criteria (CVIC) and Bayesian information criteria (BIC) were calculated to evaluate the trade-offs between model fitting and model complexities.[^20^](https://www.zotero.org/google-docs/?xZzz9I) A lower CVIC or BIC indicates a better fitted model with a low complexity i.e., a fewer number of subtypes. For each model, the probability of assigning each participant to a subtype was compared using the Wilcoxon rank sum test. The optimal number of subtypes is determined by a combination of a high log likelihood of fitting, low CVIC and BIC, and a high likelihood of assigning subtypes.

## 2. Supplementary results

### 2.1 Marker selection

The initial list of imaging markers that could be used in SuStaIn modelling was: WM volume, GM volume, ventricle volume, total WMH volume, periventricular WMH volume, deep WMH volume, lacunes, microbleed count, perivascular space volume in basal ganglia and white matter, PSMD, median MD, FA, free water (FW)[^21^](https://www.zotero.org/google-docs/?4Hciyv) in all white matter or normal appearing white matter (NAWM). NAWM was defined as white matter voxels that were not WMH. The measurement of lacune count, microbleed count and perivascular space volume was not standardized, owing to the requirement of manual counting or segmentation.

To calculate periventricular WMH volume, in the WMH mask, voxels within an Euclidean distance of 10mm from the ventricle were considered periventricular WMH.[^22^](https://www.zotero.org/google-docs/?VEared) The deep WMH volume was the total WMH volume minus the periventricular WMH volume.

The inclusion criteria for selecting markers in constructing a SuStaIn model are: 1) the marker is available across both CamcSVD and RUNDMC, 2) assessment of the marker is standardized, 3) correlation between different markers adjusted for age and sex was <0.6 in the RUNDMC cohort, 4) only imaging markers were included.[^23,24^](https://www.zotero.org/google-docs/?dQA7TQ)

In the RUNDMC dataset, periventricular WMH volume had a correlation coefficient of 0.94 with total WMH volume after adjusting for age and sex, while deep WMH volume had a correlation of 0.91. As the coefficients were > 0.6, periventricular and deep WMH volumes were not included. In the white matter region, median FA and MD had a correlation of -0.81, while median MD and FW had a correlation of 0.97. Thus, FA and FW were not included. Median MD in the white matter region had a correlation of 0.99 while MD in the normal appearing white matter. Thus, only median MD in the white matter region was included. PSMD was not included as it highly correlated with median MD. The remaining markers did not show correlations above 0.8 (**Figure S2**). Overall, five markers met the criteria for constructing SuStaIn models: ventricle volume, WM volume, GM volume, WMH volume, and median MD in all white matter.

Of note, there is significant discrepancy of PVS volumes among different cohorts within the CamcSVD dataset due to different MRI field strengths, thus precluding the use of PVS in trajectory construction. Regarding lacunes and microbleeds, as these are ordinal variables and SuStaIn requires z-transform, they cannot fit into the mathematical framework of SuStaIn.

### 2.2 Data harmonization

In the CamcSVD dataset, after ComBat harmonization, the proportion for variance explained by age, sex and WMH volume increased from 0.11 to 0.29 for PSMD, and from 0.23 to 0.26 for median MD. This indicates biological variability is preserved after harmonization. To assess the changes in cohort effects, after harmonization, no significant variance in either PSMD or median MD was found among the cohorts within the CamcSVD dataset (Barlett’s test p-value 0.948). For the cohort prediction test, after harmonization, the prediction accuracy dropped from 70.5% to 63.2%. Harmonization did not produce significant changes in PCA (**Figure S3**). Overall, ComBat harmonization reduced cohort effects while preserving biological variability of the CamcSVD dataset.

### 2.3 Selection of the optimal number of subtypes

In the RUNDMC dataset, the log likelihood of fitting significantly improved in the SuStaIn model with 2 subtypes as opposed to 1 subtype (p=0.043). The improvement is not significant when fitted with 3 subtypes (**Figure S4A**). Similarly, CVIC and BIC decreased from using 1 subtype to using 2 subtypes, but less decrease in CVIC is achieved from using 2 subtypes to using 3 subtypes while BIC increased (**Figure S4B**). Regarding the probability of assigning each participant to a particular subtype, the probability, i.e., the confidence of assigning subtype significantly decreased from using 2 subtypes to using 3 subtypes (p<0.001) (**Figure S4C**). Taken together, the model with 2 subtypes is optimal in balancing model fitting, model complexity and subtype assignment probability.

In the CamcSVD dataset, the log likelihood of fitting improved, but not significantly (p=0.11), in the SuStaIn model with 2 subtypes as opposed to 1 subtype. Less improvement is achieved when fitted with 3 subtypes (p=0.80) (**Figure S4D**). CVIC decreased from using 1 subtype to using 2 subtypes, and negligible decrease is noted in using more subtypes. BIC is lowest for the model using 2 subtypes (**Figure S4E**). The probability of assigning subtype significantly decreased from using 2 subtypes to using 3 subtypes (p<0.001) (**Figure S4F**). Taken together, the model with 2 subtypes is optimal for the CamcSVD dataset.

In the sensitivity analysis, after removing participants with gray matter atrophy and ventriculomegaly, in the RUNDMC dataset, the log likelihood of fitting significantly improved, but not significantly (p=0.052) in the SuStaIn model with 2 subtypes as opposed to 1 subtype. The improvement is not significant when fitted with 3 subtypes (p=0.579) (**Figure S9A**). Similarly, CVIC and BIC decreased from using 1 subtype to using 2 subtypes, but less decrease in CVIC is achieved from using 2 subtypes to using 3 subtypes while BIC increased (**Figure S9B**). Regarding the probability of assigning each participant to a particular subtype, the probability, i.e., the confidence of assigning subtype significantly decreased from using 2 subtypes to using 3 subtypes (p<0.001) (**Figure S9C**). Taken together, the model with 2 subtypes is optimal in balancing model fitting, model complexity and subtype assignment probability.

In the sensitivity analysis, in the CamcSVD dataset, the log likelihood of fitting improved, but not significantly (p=0.350), in the SuStaIn model with 2 subtypes as opposed to 1 subtype. Less improvement is achieved when fitted with 3 subtypes (p=0.800) (**Figure S9D**). CVIC and BIC were at the lowest when using 2 subtypes (**Figure S9E**). The probability of assigning subtype significantly decreased from using 2 subtypes to using 3 subtypes (p=0.001) (**Figure S9F**). Taken together, the model with 2 subtypes is optimal for the CamcSVD dataset.

### 2.4 Subtype assignment certainty

To evaluate the distribution of subtypes and stages, we found that subtype assignment certainty was lowest at the beginning and end of the cSVD progression trajectories (**Figure S13A, D**). Normal control was found in the early stages predominantly stage 0 and up to stage 6 (**Figure S13B, E**). Fewer participants were assigned to the late stages. At each stage, the proportion of participants assigned to subtype A remained high (**Figure S13C, F**).

## 3. Supplementary figures

**Figure S1. MRI markers of idiopathic normal pressure hydrocephalus. (A)** Evans index: maximum distance between 2 frontal horns (upper green line) divided by the maximum intracranial distance (lower green line). (**B**) Sylvian widening: proportion of cerebrospinal fluid (CSF) (marked in red) in a region encompassing the Sylvian fissure and adjacent cortices. (**C**) Temporal horn width marked by the green lines. (**D**) Proportion of non-CSF tissues (in green) in the high-convexity region. (**E**) Callosal angle: angle of the corpus callosum (marked in green) in the coronal plane passing through the posterior commissure.

**Figure S2. Correlation between different MRI metrics in the RUNDMC dataset.** Abbreviations: WM: white matter; WMH: white matter hyperintensity; GM: gray matter.

**Figure S3. Principal component (PC) analysis of different cohorts in the CamcSVD dataset before and after harmonization.**

**Figure S4. Selection of the optimal number of subtypes in SuStaIn models constructed for the RUNDMC (A-C) and CamcSVD (D-F) datasets.** (**A**) Log likelihood in the left-out datasets of 10-fold cross-validation of the RUNDMC dataset, for each SuStaIn model with a different number of subtypes. Increased log likelihood indicates a better model fit. Log likelihood of every model is compared with the model with 2 subtypes. (**B**) Bayesian information criteria (BIC) and cross validation-based information criteria (CVIC) for each model with a different number of subtypes in the RUNDMC dataset. A decreased BIC or CVIC indicates a better balance between model fit and model complexity. (**C**) Subtype assignment probability for each participant in the RUNDMC dataset. Subtype assignment probability of every model is compared with the model with 2 subtypes. (**D**) Log likelihood in the left-out datasets of 10-fold cross-validation of the CamcSVD dataset. (**E**) BIC and CVIC in the CamcSVD dataset. (**F**) Subtype assignment probability for each participant in the CamcSVD dataset. (**G**) Log likelihood in the left-out datasets of 10-fold cross-validation of the RUNDMC dataset. P-values from Wilcoxon rank sum test were coded as: ns: >0.05, *: 0.01-0.05, **: 0.001-0.01, ***: <0.001, ****: <0.0001.

**Figure S5. Positional variance diagram of each subtype in each dataset.** Each row corresponds to one imaging feature. Along the stages of cSVD progression, when an imaging marker reaches a particular z-score, it is displayed in either red (z-score=1), purple (z-score=2) or blue color (z-score=3). For example, in figure 1A, for subtype A, the marker PSMD reaches high abnormality (z-score=3) at an early stage (stage=5).

**Figure S6**. **Correlation of the ordering of imaging features between different datasets.** Each point corresponds to the stage when an imaging marker reaches a particular z-score. The color of each point denotes different imaging markers, while the shape represents a particular z-score. **Abbreviations**: GM: gray matter, WM: white matter, WMH: white matter hyperintensity, MD: mean diffusivity.

**Figure S7. Staging consistency in longitudinal follow-up of participants in RUNDMC (A-B) and CamcSVD datasets (C-D)**. (**A**) SuStaIn stages of participants at baseline visit (visit 0) versus SuStaIn stages of the same participants at the first follow up (visit 1) in the RUNDMC dataset. Dashed line represents the points where SuStaIn stages at the two visits were identical. Dots above the dashed line means the stages at visit 1 were greater than visit 0. (**B**) SuStaIn stages of participants at visit 1 versus SuStaIn stages of the same participants at the second follow up (visit 2) in the RUNDMC dataset. (**C**) SuStaIn stages of participants at visit 0 versus SuStaIn stages of the same participants at visit 1 in the CamcSVD dataset. (**D**) SuStaIn stages of participants at visit 1 versus SuStaIn stages of the same participants at visit 2 in the CamcSVD dataset.

**Figure S8. Consistency of SuStaIn subtypes in RUNDMC** **and** **CamcSVD**. The percentage of participants whose subtype assignment was stable and whose subtype classification switched, from visit 0 to visit 1, and from visit 1 to visit 2.

**Figure S9. Selection of the optimal number of subtypes in SuStaIn models constructed for the RUNDMC (A-C) and CamcSVD (D-F) datasets in sensitivity analysis.** (**A**) Log likelihood in the left-out datasets of 10-fold cross-validation of the RUNDMC dataset, for each SuStaIn model with a different number of subtypes. Increased log likelihood indicates a better model fit. Log likelihood of every model is compared with the model with 2 subtypes. (**B**) Bayesian information criteria (BIC) and cross validation-based information criteria (CVIC) for each model with a different number of subtypes in the RUNDMC dataset. A decreased BIC or CVIC indicates a better balance between model fit and model complexity. (**C**) Subtype assignment probability for each participant in the RUNDMC dataset. Subtype assignment probability of every model is compared with the model with 2 subtypes. (**D**) Log likelihood in the left-out datasets of 10-fold cross-validation of the CamcSVD dataset. (**E**) BIC and CVIC in the CamcSVD dataset. (**F**) Subtype assignment probability for each participant in the CamcSVD dataset. (**G**) Log likelihood in the left-out datasets of 10-fold cross-validation of the RUNDMC dataset. P-values from Wilcoxon rank sum test were coded as: ns: >0.05, *: 0.01-0.05, **: 0.001-0.01, ***: <0.001, ****: <0.0001.

**Figure S10**. **Staging consistency of each participant in sensitivity analysis**. Comparison of the assigned SuStaIn stages in the full datasets, with the subsets after removing patients with ventriculomegaly and gray matter atrophy.

**Figure S11**. **Staging consistency of each biomarker in sensitivity analysis**. Each point corresponds to the stage when an imaging marker reaches a particular z-score. The color of each point denotes different imaging markers, while the shape represents a particular z-score.

**Figure S12**: **cSVD scores along SuStaIn stages.**

**Figure S13. Details of subtype and stage assignment.** Each row of figures pertains to results obtained from RUNDMC or CamcSVD. (**A**) Probability of the most likely subtype in each stage in RUNDMC. (**B**) Number of normal control and cSVD patients in each stage in RUNDMC. (**C**) Number of subjects assigned to each subtype in each stage in RUNDMC. (**D**) Probability of the most likely subtype in each stage in CamcSVD. (**E**) Number of normal control and cSVD patients in each stage in CamcSVD. (**F**) Number of subjects assigned to each subtype in each stage in CamcSVD.

## 4. Supplementary Table

| **Criteria** | **MINERVA** | **NETWORKS** | **PRESERVE** | | **SCANS** | | | | **RUNDMC** | | |
| --- | --- | --- | --- | --- | --- | --- | --- | --- | --- | --- | --- |
|  | **TP0** | **TP0** | **TP0** | **TP1** | **TP0** | **TP1** | **TP2** | **TP3** | **TP0** | **TP1** | **TP2** |
| Initial cohort size | 73 | 46 | 111 | 84 | 121 | 96 | 74 | 68 | 503 | 357 | 294 |
| Exclude participants without T1, FLAIR or DTI, lacune or microbleed data | 73 | 45 | 106 | 84 | 104 | 96 | 74 | 67 | 496 | 354 | 281 |
| Exclude participants with corrupted image files | 73 | 45 | 105 | 77 | 104 | 96 | 74 | 67 | 496 | 354 | 281 |
| Exclude participants with motion artifacts | 73 | 45 | 105 | 77 | 104 | 96 | 74 | 66 | 496 | 354 | 281 |
| Exclude participants overlapped across datasets | 73 | 33 | 105 | 77 | 104 | 96 | 74 | 66 | 496 | 354 | 281 |
| Exclude participants missing demographics | 73 | 33 | 104 | 77 | 104 | 96 | 74 | 66 | 496 | 354 | 281 |
| Final cohort size | 73 | 33 | 104 | 77 | 104 | 96 | 74 | 66 | 496 | 354 | 281 |

**Table S1**. **Cohort selection.** The MINERVA,[^25^](https://www.zotero.org/google-docs/?L72WlV) MRC-PET,[^26^](https://www.zotero.org/google-docs/?7dceK2) NETWORKS,[^27^](https://www.zotero.org/google-docs/?6BgVHN) PRESERVE[^28^](https://www.zotero.org/google-docs/?UvQkt7) and SCANS[^29^](https://www.zotero.org/google-docs/?wMidFB) cohorts together make up the CamcSVD dataset. We grouped participants from the MINERVA study and MRC-PET study as the “MINERVA cohort”, given their similar sequence designs and inclusion criterias. The MINERVA cohort has 19 normal controls while the NETWORKS cohort has 12 normal controls. **Abbreviations**: TP: time point.

| **Sequence** | **Parameter** | **MINERVA (3T)** | **NETWORKS (3T)** | **SCANS (1.5T)** | **RUNDMC (1.5T)** |
| --- | --- | --- | --- | --- | --- |
| T1 | TR(ms) |  | 2300 | 11.5 | 22.5 |
|  | TE(ms) |  | 2.98 | 5 | 3.68 |
|  | Slice(mm) | 1 | 1 | 1.1 | 1 |
| FLAIR | TR(ms) | 8800 | 12540 | 9000 | 9000 |
|  | TE(ms) | 120 | 132 | 130 | 84 |
|  | Slice(mm) | 5 | 2 | 5 | 3 |
| dMRI | TR(ms) | 15763 | 11700 | 15600 | 10200 |
|  | TE(ms) |  | 106 | 93.4 | 95 |
|  | Slice(mm) | 2 | 2 | 2.5 | 2 |
|  | b-values(s/mm^2^) | 1000 | 1000 | 1000 | 900 |
|  | b0 volumes | 5 | 4 | 1 | 4 |
|  | RPE volumes | 0 | 0 | 0 | 0 |
|  | Directions | 63 | 59 | 25 | 61 |

**Table S2**. **MRI sequence parameters. Abbreviations**: RPE: reverse phase encodings, TR: repetition time, TE: echo time.

| **Sequence** | **Parameter** | **Site 1** | **Site 2** | **Site 3** | **Site 4** | **Site 5** | **Site 6** |
| --- | --- | --- | --- | --- | --- | --- | --- |
| T1 | TR(ms) | 8.24 | 9.81 | 2200 | 11 | 8.53 | 2200 |
|  | TE(ms) | 4.61 | 4.60 | 2.97 | 4.61 | 4.61 | 2.94 |
|  | Slice(mm) | 1 | 1 | 1 | 1 | 1 | 1 |
| FLAIR | TR(ms) | 11000 | 11000 | 8000 | 11000 | 11000 | 8000 |
|  | TE(ms) | 120 | 120 | 124 | 120 | 120 | 121 |
|  | Slice(mm) | 3 | 3 | 3 | 3 | 3 | 3 |
| DTI | TR(ms) | 6850 | 6850 | 6850 | 6850 | 9100 | 11500 |
|  | TE(ms) | 75 | 75 | 75 | 75 | 82 | 93 |
|  | Slice(mm) | 2 | 2 | 2 | 2 | 2 | 2 |
|  | b-values(s/mm^2^ ) | 1000 | 1000 | 1000 | 1000 | 1000 | 1000 |
|  | b0 volumes | 1 | 1 | 1 | 1 | 1 | 1 |
|  | RPE volumes | 0 | 0 | 0 | 0 | 0 | 0 |
|  | Directions | 32 | 32 | 32 | 32 | 32 | 32 |

**Table S3**. **PRESERVE MRI sequence parameters across all sites. Abbreviations**: RPE: reverse phase encodings, TR: repetition time, TE: echo time.

| **Feature** | **Test** | **Description** |
| --- | --- | --- |
| Executive function | Inhibition | Divide the Stroop part III SAT score by the mean of the SAT score of parts I and II[^30,31^](https://www.zotero.org/google-docs/?SW8ygZ) |
|  | VSAT | Verbal Series Attention Test: forward and reverse generation of arithmetic series; number-letter sequencing; auditory vigilance[^32^](https://www.zotero.org/google-docs/?F42Fwn) |
|  | Verbal fluency | Naming animals and professions |
|  | Processing speed |  |
| Processing speed | PPMS | Pencil-pen memory scanning[^33^](https://www.zotero.org/google-docs/?RLv73G) |
|  | Stroop test | Color word test[^30,31^](https://www.zotero.org/google-docs/?P8tjLd) |
|  | SDST | Symbol–Digit Substitution Task involving match letters to numbers[^34^](https://www.zotero.org/google-docs/?GCOlZE) according to a key |
| Long term memory | RALVT | Rey Auditory Verbal Learning Test[^35^](https://www.zotero.org/google-docs/?w2gtVr) |
|  | RCFT | Rey Complex Figure Task involves reproducing a complicated line drawing, first by copying it freehand, and drawing from memory[^35^](https://www.zotero.org/google-docs/?qvg6el) |

**Table S4**. Cognitive testing in the RUNDMC dataset.

| **Features** | **RUNDMC (n=496)** | **CamcSVD (n=330)** |
| --- | --- | --- |
| Tissue volume | |  |
| Raw GM volume (mL) | 0.00 | 0.00 |
| Raw WM volume (mL) | 0.00 | 0.00 |
| Raw ventricle volume (mL) | 0.00 | 0.00 |
| cSVD markers | |  |
| Raw WMH volume (mL) | 0.00 | 0.00 |
| PSMD (10^-3^ mm^2^/s) | 0.00 | 0.00 |
| Median MD (10^-3^ mm^2^/s) | 0.00 | 0.00 |
| CV risk factors | |  |
| Smoking/Smoker | 0.30 | 0.00 |
| Diabetes | 0.00 | 0.00 |
| Hypertension | 0.00 | 0.04 |
| CSF dynamic markers | |  |
| PVS volume BG (mm^3^) | 0.00 | 0.64 |
| PVS volume WM (mm^3^) | 0.00 | 0.64 |
| DTI-ALPS | 0.00 | 0.00 |
| iNPH markers | |  |
| Evans index | 0.00 | 0.01 |
| Temporal horn width | 0.00 | 0.01 |
| Sylvian widening | 0.00 | 0.01 |
| High convexity tightness | 0.00 | 0.01 |
| Callosal angle | 0.00 | 0.01 |
| Cognition | |  |
| Long term memory | 0.00 | - |
| executive function | 0.00 | - |
| Processing speed | 0.00 | - |

**Table S5**. **Percentage of data missing for each feature at the baseline visit of each dataset. Abbreviations**: BG: basal ganglia, GM: gray matter, WM: white matter, WMH: White matter hyperintensity, PSMD: peak width of skeletonized mean diffusivity, MD: mean diffusivity, CV: cardiovascular, iNPH: idiopathic normal pressure hydrocephalus.

|  | **RUNDMC (n=496)** | **CamcSVD (n=330)** | **p** |
| --- | --- | --- | --- |
| Demographics | | | |
| Age (Years), median (IQR) | 64.8 (58.1-73.0) | 70.9 (64.0-76.9) | <0.001 |
| Sex (Males), n(%) | 280 (56.5) | 206 (62.4) | <0.001 |
| Education (Years), median (IQR) | 10.0 (8.0-15.0) | 12.0 (10.0-14.0) | 0.001 |
| Tissue volumes | | | |
| Raw GM volume (mL), median (IQR) | 701.1 (659.8-739.0) | 679.9 (630.4-728.1) | 0.01 |
| Raw WM volume (mL), median (IQR) | 512.6 (475.9-551.3) | 499.3 (456.3-543.7) | 0.556 |
| Raw ventricle volume (mL), median (IQR) | 35.6 (24.4-53.5) | 39.3 (28.2-57.9) | 0.550 |
| cSVD markers | | | |
| Raw WMH volume (mL), median (IQR) | 2.5 (1.0-7.5) | 21.8 (11.9-38.9) | <0.001 |
| Median MD (10^-3^ mm^2^/s), median (IQR) | 0.8 (0.8-0.8) | 0.8 (0.8-0.8) | <0.001 |
| Microbleeds, median (IQR) | 0.0 (0.0-0.0) | 0.0 (0.0-2.0) | <0.001 |
| Lacunes, median (IQR) | 0.0 (0.0-1.0) | 2.0 (1.0-5.0) | <0.001 |
| CV risk factors | | | |
| Smoker, n(%) | 75 (21.5) | 179 (54.4) | <0.001 |
| Diabetes, n(%) | 64 (12.9) | 63 (19.1) | <0.001 |
| Hypertension, n(%) | 246 (49.6) | 228 (71.9) | 0.222 |
| CSF dynamic markers | | | |
| PVS volume BG (mm^3^), median (IQR) | 157.5 (95.0-262.5) | - | - |
| PVS volume WM (mm^3^), median (IQR) | 994.5 (607.5-1728.2) | - | - |
| DTI-ALPS | 1.2 (1.0-1.3) | 1.3 (1.1-1.4) | 0.102 |
| iNPH markers | | | |
| Evans index | 0.3 (0.3-0.3) | 0.3 (0.3-0.3) | <0.001 |
| Temporal horn width | 4.3 (3.6-5.3) | 4.5 (3.7-5.4) | <0.001 |
| Sylvian widening | 0.2 (0.2-0.2) | 0.2 (0.2-0.2) | <0.001 |
| High convexity tightness | 0.8 (0.7-0.8) | 0.7 (0.7-0.8) | 0.017 |
| Callosal angle (^0^), median (IQR) | 112.2 (98.5-119.7) | 111.7 (101.1-121.5) | <0.001 |
| Cognitive markers | | | |
| Memory (z-score), median (IQR) | -0.2 (-1.1-0.6) | - | - |
| Executive function (z-score), median (IQR) | -0.3 (-1.0-0.4) | - | - |
| Processing speed (z-score), median (IQR) | -0.5 (-1.2-0.3) | - | - |

**Table S6. Baseline demographics**. Lower cognitive markers mean worse cognition. P-values of comparing categorical variables were derived from the chi-squared test. P-values of comparing continuous variables were derived from the Wilcoxon rank-sum test. PVS volumes and cognitive markers in the CamcSVD dataset were not reported owing to differing measures across the constituent cohorts within the CamcSVD dataset. **Abbreviations**: IQR: interquartile range, BG: basal ganglia, GM: gray matter, WM: white matter, WMH: White matter hyperintensity, MD: mean diffusivity.

| **Dataset** | **Percentage of radiologically probable iNPH** | | **RR (95% CI)** | **p** |
| --- | --- | --- | --- | --- |
|  | **Subtype A** | **Subtype B** |  |  |
| RUNDMC | 0.130 | 0.413 | 3.18 (2.25-4.49) | p<0.001 |
| CamcSVD | 0.123 | 0.438 | 3.55 (2.43-5.19) | p<0.001 |

**Table S7**. **Percentage of radiologically probable iNPH in each cSVD subtype in each dataset.** P-values were calculated using the Fisher's exact test.

| **Dataset** |  | **Original subtype assignment** | |
| --- | --- | --- | --- |
|  | **Sensitivity analysis** | **Subtype A** | **Subtype B** |
| RUNDMC | Subtype A | 383 | 15 |
|  | Subtype B | 50 | 360 |
| CamcSVD | Subtype A | 407 | 0 |
|  | Subtype B | 11 | 97 |

**Table S8**: **Stability of subtype assignment in sensitivity analysis.** Comparison of the assigned subtypes in the full datasets, with the subsets after removing patients with ventriculomegaly and gray matter atrophy.

## References

[1.](https://www.zotero.org/google-docs/?4iuCKf)	[Avants, B. B., Tustison, N. & Johnson, H. Advanced Normalization Tools (ANTS). (2014).](https://www.zotero.org/google-docs/?4iuCKf)

[2.](https://www.zotero.org/google-docs/?4iuCKf)	[Isensee, F. *et al.* Automated brain extraction of multisequence MRI using artificial neural networks. *Human Brain Mapping* **40**, 4952–4964 (2019).](https://www.zotero.org/google-docs/?4iuCKf)

[3.](https://www.zotero.org/google-docs/?4iuCKf)	[Schilling, K. G. *et al.* Synthesized b0 for diffusion distortion correction (Synb0-DisCo). *Magn Reson Imaging* **64**, 62–70 (2019).](https://www.zotero.org/google-docs/?4iuCKf)

[4.](https://www.zotero.org/google-docs/?4iuCKf)	[Billot, B. *et al.* SynthSeg: Segmentation of brain MRI scans of any contrast and resolution without retraining. *Medical Image Analysis* **86**, 102789 (2023).](https://www.zotero.org/google-docs/?4iuCKf)

[5.](https://www.zotero.org/google-docs/?4iuCKf)	[Mojiri Forooshani, P. *et al.* Deep Bayesian networks for uncertainty estimation and adversarial resistance of white matter hyperintensity segmentation. *Human Brain Mapping* **43**, 2089–2108 (2022).](https://www.zotero.org/google-docs/?4iuCKf)

[6.](https://www.zotero.org/google-docs/?4iuCKf)	[Woolrich, M. W. *et al.* Bayesian analysis of neuroimaging data in FSL. *Neuroimage* **45**, S173-186 (2009).](https://www.zotero.org/google-docs/?4iuCKf)

[7.](https://www.zotero.org/google-docs/?4iuCKf)	[Chen, Y., Hong, H., Nazeri, A., Markus, H. S. & Luo, X. Cerebrospinal fluid-based spatial statistics: towards quantitative analysis of cerebrospinal fluid pseudodiffusivity. *Fluids Barriers CNS* **21**, 59 (2024).](https://www.zotero.org/google-docs/?4iuCKf)

[8.](https://www.zotero.org/google-docs/?4iuCKf)	[Baykara, E. *et al.* A Novel Imaging Marker for Small Vessel Disease Based on Skeletonization of White Matter Tracts and Diffusion Histograms. *Annals of Neurology* **80**, 581–592 (2016).](https://www.zotero.org/google-docs/?4iuCKf)

[9.](https://www.zotero.org/google-docs/?4iuCKf)	[Hong, H. *et al.* Perivascular space dysfunction in cerebral small vessel disease is related to neuroinflammation. *Brain* awae357 (2024) doi:10.1093/brain/awae357.](https://www.zotero.org/google-docs/?4iuCKf)

[10.](https://www.zotero.org/google-docs/?4iuCKf)	[Benveniste, H. & Nedergaard, M. Cerebral small vessel disease: A glymphopathy? *Curr Opin Neurobiol* **72**, 15–21 (2022).](https://www.zotero.org/google-docs/?4iuCKf)

[11.](https://www.zotero.org/google-docs/?4iuCKf)	[Huang, P. *et al.* Development and validation of a perivascular space segmentation method in multi-center datasets. *Neuroimage* **298**, 120803 (2024).](https://www.zotero.org/google-docs/?4iuCKf)

[12.](https://www.zotero.org/google-docs/?4iuCKf)	[Taoka, T. *et al.* Evaluation of glymphatic system activity with the diffusion MR technique: diffusion tensor image analysis along the perivascular space (DTI-ALPS) in Alzheimer’s disease cases. *Jpn J Radiol* **35**, 172–178 (2017).](https://www.zotero.org/google-docs/?4iuCKf)

[13.](https://www.zotero.org/google-docs/?4iuCKf)	[Cai, H. *et al.* The contribution of cerebral small vessel disease in idiopathic normal pressure hydrocephalus: Insights from a prospective cohort study. *Alzheimer’s & Dementia* **21**, e14395 (2025).](https://www.zotero.org/google-docs/?4iuCKf)

[14.](https://www.zotero.org/google-docs/?4iuCKf)	[Lee, J. *et al.* Automated Idiopathic Normal Pressure Hydrocephalus Diagnosis via Artificial Intelligence–Based 3D T1 MRI Volumetric Analysis. *American Journal of Neuroradiology* **46**, 33–40 (2025).](https://www.zotero.org/google-docs/?4iuCKf)

[15.](https://www.zotero.org/google-docs/?4iuCKf)	[Ardekani, B. A. & Bachman, A. H. Model-based automatic detection of the anterior and posterior commissures on MRI scans. *Neuroimage* **46**, 677–682 (2009).](https://www.zotero.org/google-docs/?4iuCKf)

[16.](https://www.zotero.org/google-docs/?4iuCKf)	[Walt, S. van der *et al.* scikit-image: image processing in Python. *PeerJ* **2**, e453 (2014).](https://www.zotero.org/google-docs/?4iuCKf)

[17.](https://www.zotero.org/google-docs/?4iuCKf)	[Constantinescu, C. *et al.* Prevalence of Possible Idiopathic Normal Pressure Hydrocephalus in Sweden. *Neurology* **102**, e208037 (2024).](https://www.zotero.org/google-docs/?4iuCKf)

[18.](https://www.zotero.org/google-docs/?4iuCKf)	[Chen, Y. *et al.* Improved Dementia Prediction in Cerebral Small Vessel Disease Using Deep Learning–Derived Diffusion Scalar Maps From T1. *Stroke* **55**, 2254–2263 (2024).](https://www.zotero.org/google-docs/?4iuCKf)

[19.](https://www.zotero.org/google-docs/?4iuCKf)	[Fortin, J.-P. *et al.* Harmonization of multi-site diffusion tensor imaging data. *Neuroimage* **161**, 149–170 (2017).](https://www.zotero.org/google-docs/?4iuCKf)

[20.](https://www.zotero.org/google-docs/?4iuCKf)	[Chen, H. *et al.* Transferability of Alzheimer’s disease progression subtypes to an independent population cohort. *NeuroImage* **271**, 120005 (2023).](https://www.zotero.org/google-docs/?4iuCKf)

[21.](https://www.zotero.org/google-docs/?4iuCKf)	[Maillard, P. *et al.* Cerebral white matter free water: A sensitive biomarker of cognition and function. *Neurology* **92**, e2221–e2231 (2019).](https://www.zotero.org/google-docs/?4iuCKf)

[22.](https://www.zotero.org/google-docs/?4iuCKf)	[Griffanti, L. *et al.* Classification and characterization of periventricular and deep white matter hyperintensities on MRI: A study in older adults. *NeuroImage* **170**, 174–181 (2018).](https://www.zotero.org/google-docs/?4iuCKf)

[23.](https://www.zotero.org/google-docs/?4iuCKf)	[Young, A. L. *et al.* Uncovering the heterogeneity and temporal complexity of neurodegenerative diseases with Subtype and Stage Inference. *Nat Commun* **9**, 4273 (2018).](https://www.zotero.org/google-docs/?4iuCKf)

[24.](https://www.zotero.org/google-docs/?4iuCKf)	[Young, A. L. *et al.* Disease Progression Modeling in Chronic Obstructive Pulmonary Disease. *Am J Respir Crit Care Med* **201**, 294–302 (2020).](https://www.zotero.org/google-docs/?4iuCKf)

[25.](https://www.zotero.org/google-docs/?4iuCKf)	[Brown, R. B. *et al.* MINocyclinE to Reduce inflammation and blood brain barrier leakage in small Vessel diseAse (MINERVA) trial study protocol. *Eur Stroke J* **7**, 323–330 (2022).](https://www.zotero.org/google-docs/?4iuCKf)

[26.](https://www.zotero.org/google-docs/?4iuCKf)	[Walsh, J. *et al.* Microglial activation and blood–brain barrier permeability in cerebral small vessel disease. *Brain* **144**, 1361–1371 (2021).](https://www.zotero.org/google-docs/?4iuCKf)

[27.](https://www.zotero.org/google-docs/?4iuCKf)	[Lawrence, A. J., Tozer, D. J., Stamatakis, E. A. & Markus, H. S. A comparison of functional and tractography based networks in cerebral small vessel disease. *Neuroimage Clin* **18**, 425–432 (2018).](https://www.zotero.org/google-docs/?4iuCKf)

[28.](https://www.zotero.org/google-docs/?4iuCKf)	[Croall, I. D. *et al.* Using DTI to assess white matter microstructure in cerebral small vessel disease (SVD) in multicentre studies. *Clinical Science* **131**, 1361–1373 (2017).](https://www.zotero.org/google-docs/?4iuCKf)

[29.](https://www.zotero.org/google-docs/?4iuCKf)	[Zeestraten, E. A. *et al.* Application of Diffusion Tensor Imaging Parameters to Detect Change in Longitudinal Studies in Cerebral Small Vessel Disease. *PLOS ONE* **11**, e0147836 (2016).](https://www.zotero.org/google-docs/?4iuCKf)

[30.](https://www.zotero.org/google-docs/?4iuCKf)	[Stroop, J. R. Studies of interference in serial verbal reactions. *Journal of Experimental Psychology* **18**, 643–662 (1935).](https://www.zotero.org/google-docs/?4iuCKf)

[31.](https://www.zotero.org/google-docs/?4iuCKf)	[Jensen, A. R. & Rohwer, W. D. The Stroop color-word test: a review. *Acta Psychol (Amst)* **25**, 36–93 (1966).](https://www.zotero.org/google-docs/?4iuCKf)

[32.](https://www.zotero.org/google-docs/?4iuCKf)	[Mahurin, R. K. & Cooke, N. Verbal series attention test: Clinical utility in the assessment of dementia. *The Clinical Neuropsychologist* **10**, 43–53 (1996).](https://www.zotero.org/google-docs/?4iuCKf)

[33.](https://www.zotero.org/google-docs/?4iuCKf)	[Brand, N. & Jolles, J. Information processing in depression and anxiety. *Psychol Med* **17**, 145–153 (1987).](https://www.zotero.org/google-docs/?4iuCKf)

[34.](https://www.zotero.org/google-docs/?4iuCKf)	[van der Elst, W., van Boxtel, M. P. J., van Breukelen, G. J. P. & Jolles, J. The Letter Digit Substitution Test: normative data for 1,858 healthy participants aged 24-81 from the Maastricht Aging Study (MAAS): influence of age, education, and sex. *J Clin Exp Neuropsychol* **28**, 998–1009 (2006).](https://www.zotero.org/google-docs/?4iuCKf)

[35.](https://www.zotero.org/google-docs/?4iuCKf)	[Rey, A. L’examen psychologique dans les cas d’encéphalopathie traumatique. (Les problems.). in (1941).](https://www.zotero.org/google-docs/?4iuCKf)
